# Surgical Site Infections Following Spine Surgery: Eliminating the Controversies in the Diagnosis

**DOI:** 10.3389/fmed.2014.00007

**Published:** 2014-03-24

**Authors:** Jad Chahoud, Zeina Kanafani, Souha S. Kanj

**Affiliations:** ^1^Division of Infectious Diseases, Department of Internal Medicine, American University of Beirut Medical Center, Beirut, Lebanon

**Keywords:** post-surgical spine infection, post-procedural discitis, imaging, risk factors, *Staphylococcus aureus*, inflammatory markers

## Abstract

Surgical site infection (SSI) following spine surgery is a dreaded complication with significant morbidity and economic burden. SSIs following spine surgery can be superficial, characterized by obvious wound drainage or deep-seated with a healed wound. *Staphylococcus aureus* remains the principal causal agent. There are certain pre-operative risk factors that increase the risk of SSI, mainly diabetes, smoking, steroids, and peri-operative transfusions. Additionally, intra-operative risk factors include surgical invasiveness, type of fusion, implant use, and traditional instead of minimally invasive approach. A high level of suspicion is crucial to attaining an early definitive diagnosis and initiating appropriate management. The most common presenting symptom is back pain, usually manifesting 2–4 weeks and up to 3 months after a spinal procedure. Scheduling a follow-up visit between weeks 2 and 4 after surgery is therefore necessary for early detection. Inflammatory markers are important diagnostic tools, and comparing pre-operative with post-operative levels should be done when suspecting SSIs following spine surgery. Particularly, serum amyloid A is a novel inflammatory marker that can expedite the diagnosis of SSIs. Magnetic resonance imaging remains the diagnostic modality of choice when suspecting a SSI following spine surgery. While 18F-fluorodeoxyglucose-positron emission tomography is not widely used, it may be useful in challenging cases. Despite their low yield, blood cultures should be collected before initiating antibiotic therapy. Samples from wound drainage should be sent for Gram stain and cultures. When there is a high clinical suspicion of SSI and in the absence of superficial wound drainage, computed tomography-guided aspiration of paraspinal collections is warranted. Unless the patient is hemodynamically compromised, antibiotics should be deferred until proper specimens for culture are secured.

## Introduction

Surgical site infections (SSIs) following spine surgery comprise superficial and deep infections and were first described as a clinical entity by Turnbull in 1953 (1). Superficial spine infections are localized to the skin and subcutaneous tissue. On the other hand, deep infections disseminate under the fascia and encompass discitis, epidural abscess, and spondylitis; this type of infections is characterized by inflammation of the intervertebral disks and associated soft and articular tissues (2).

Although SSIs following spine surgery can be prevented to a great extent using general measures intended to avert all potential SSIs, they remain a dreaded complication. Some of these general preventive approaches include adoption of aseptic techniques, optimization of patient status pre-operatively as well as intra-operatively, appropriate use of pre-operative antibiotics, and good post-operative follow-up (3–6). SSIs result in significant increase in morbidity and incur a substantial cost to the health care system (7). In one study, each episode of wound infection following spine procedure contributed to a mean increase in the cost of care by $4,067 (CI, $1,682.79–6,872.39); (*P* = 0.0004) compared to a non-complicated case (8). A high index of suspicion is compulsory in every patient presenting with back pain after any invasive diagnostic or therapeutic spinal procedure (9). Physicians may sometimes struggle with diagnosing SSI due to a number of difficulties, including the paucity of physical examination findings, mimicry of non-infective conditions, presence of minor symptoms leading to patients not seeking medical attention, inadequate follow-up strategies in some institutions, and the previous dependence on plain X-rays that lack sensitivity for diagnosing SSI (10).

However, the use of a well-defined systematic approach would help in establishing a definitive diagnosis in a timely manner. This would be based on a comprehensive history, thorough physical examination, detailed laboratory studies, blood cultures, and cultures of wound or computed tomography (CT)-guided aspirate material, and imaging studies.

In this paper, we attempt to provide a comprehensive review on the diagnostic approach to SSIs following spine surgery. We discuss the incidence of SSIs based on type of procedure, detailing the factors that place patients at increased risk for infection and highlighting the spectrum of involved pathogens. We also review clinical, laboratory, and imaging techniques in the diagnosis of SSI, addressing some of the controversies. Treatment of SSIs is beyond the scope of this article and will not be reviewed here.

## Epidemiology

The reported incidence of SSIs following spine surgery ranges from 0.5 to 18.8% (11–19). Such wide-ranging results from different reports are most probably due to significant variations in operative factors such as the use of implants, case complexity, and the surgical approach itself. Additionally, in some cases, the discitis may be self-limited and may not be reported to the surgeon, whereas in other cases, patients may suffer from fulminant sepsis with abscess development.

A crucial need exists for documentation of an exact incidence of SSIs at every center depending on the surgical procedure. This will direct pre-operative patient counseling, improve quality of care, enhance the effectiveness of infection control measures, and potentially alleviate medico-legal concerns.

Table 1 summarizes studies that have looked at the incidence of SSI following spine surgery and that provided data on type of surgery and type of infection. The largest study by Smith et al. included a total of 108,419 patients from 2004 to 2007. The primary endpoint of the study was to estimate the incidence of SSIs and the secondary endpoint was to assess risk factors for infection (20). Infections were deemed to be superficial in 0.8% and deep in 1.3% of cases. Other studies in heterogeneous surgical conditions between 2001 and 2012 (Table 1) reported infection rates from 0.15 to 7.2% (20–33).

**Table 1:** **Prospective and retrospective clinical studies on incidence of SSI incidence following spine surgery (20–33)**.

Reference	Study design	Type of interventional procedures (%)	Type of SSI considered in the study	Number of total study patients	SSI rate (%)
(20)	Retrospective review of prospectively collected data	Invasive (87); minimally invasive (13)	Superficial or deep	108,419	6.7
(21)	Retrospective review of prospectively collected data	Decompressive (78); instrumented (20); intra-dural (2)	Superficial or deep	1,274	0.22
(22)	Retrospective data review	Decompressive (89); instrumented (1.4)	Superficial or deep	663	0.15
(23)	Retrospective case–control study	Decompressive (27.4); instrumented (72.5)	Superficial or deep	2,316	2.0
(25)	Retrospective data review	Instrumented fusions (100)	Deep only	1,980	3.7
(24)	Case–control study	Laminectomy (100)	Superficial or deep	6,365	1.0
(28)	Retrospective data review	Instrumented posterior (82); anterior (18)	Deep only	326	4.3
(26)	Retrospective data review	Decompressive (60); instrumented (40)	Deep only	1,133	0.7
(27)	Retrospective data review	Instrumented posterior interbody fusion (100)	Deep only	111	7.2
(33)	Prospective case–control study	Mixed decompressive and instrumented	Superficial or deep	997	2.7
(29)	Retrospective case–control	Mixed decompressive and instrumented	Deep only	1,095	4.4
(30)	Retrospective case–control study	Mixed decompressive and instrumented	Superficial or deep	1,918	2.8
(31)	Prospective surveillance study	Laminectomy	Nosocomial infection	37,137	0.9–2.6
(31)	Prospective surveillance study	Spinal fusion	Nosocomial infection	21,491	1.2–7.2
(32)	Retrospective data review	Instrumental lumbar fusion (100)	Deep only	817	3.2
(34)	Retrospective data review	Mixed decompressive and instrumented	Deep only	2,391	1.9

Infection rates vary greatly according to type of initial surgical intervention (Table 2) (16, 20, 34). Notably, surgeries for spinal trauma are associated with the highest SSI rates, reported to be 9.4% in one study (20). Patients undergoing spine surgeries for metastatic tumor and acute osteodiscitis constitute two other subgroups with high infection rates of around 5%. On the other hand, surgeries for degenerative disease have the lowest reported infection rate of 1.4%. Moreover, minimally invasive spinal procedures seem to be associated with a much lower infection rate than open procedures (0.5 vs. 2.4%, *P* = 0.001) (20). In the study by Smith et al., the overall rate of infection among adults varied depending on the location of spine surgery. The highest rates were for thoracic procedures (2.1%), followed by lumbar (1.6%) and cervical procedures (0.8%) (20). Other factors that affect infection rates include the nature of the surgical procedures. Spinal fusion had a 33% higher risk of infection than procedures without fusion (20). In addition, infections rates vary depending on the approach to spinal fusion. For instance, cases with anterior fusion only showed significantly lower infection rates (0.6%) compared to the overall rate of infection associated with fusion cases. This lower infection rate with the anterior approach may be explained by less extensive muscle dissection for bone exposure and the better vascularity of the anterior spine. For the other types of fusion, significantly higher infection rates were reported. These included combined anterior–posterior fusions (3.2%), posterolateral-only fusions (3.0%), and interlaminar facet-only fusions (2.8%) (20).

**Table 2:** **Rate of SSI following spine surgery by type of surgery**.

Type of surgery (reference number)	Rate of SSI (%)
Trauma (20)	9.4
Acute discitis (20)	5.1
Metastatic tumor (20, 34)	5.1
Kyphosis (20)	4.2
Scoliosis (20)	3.7
Elective spinal surgery (20)	3.7
Implant revision (20)	3.2
Non-minimally invasive (20)	2.4
Degenerative disease (20)	1.4
Minimally invasive approach (16, 20)	0.5

The presence of implants is another factor that significantly increases SSI risk. Cases with instrumentation have resulted in a 28% higher infection rates than cases without implants (20). Implants provide an avascular surface for the bacteria to form a biofilm and constitute a nidus for microbial growth, hence escaping antibiotic activity and the host immune system.

In addition, revision cases are associated with a 65% higher rate of infection compared with primary cases. This significant increase in infection rates was even more evident for deep wound (2.2 vs. 1.2%) compared with superficial wound infections (1.1 vs. 0.8%) (20).

## Specific Risk Factors for SSI Development

The incidence of SSIs is determined by both pre-operative and intra-operative risk factors. Several pre-operative patient factors (Table 3) have been incriminated in significantly increasing SSI risk. Diabetes (35) and cigarette smoking (28) for instance, are both associated with tissue ischemia and small vessel damage, predisposing to increased risk of infection. In addition, obesity constitutes a risk factor for SSI due to the thick layer of adipose tissue in obese patients, characterized by poor perfusion and presenting a large space for potential infective processes (28). Other identified peri-operative risk factors for SSI include steroid use (23, 36), alcohol abuse (28, 37), extremes of ages (28, 36, 37), and transfusion of blood products (38).

**Table 3:** **Surgical site infection pre-operative risk factors (28, 34–38)**.

Diabetes
Cigarette smoking
Obesity
Steroid use
Alcohol abuse
Extremes of ages
Peri-operative transfusion of blood products

In addition to pre-operative risk factors, multiple surgical factors have been assessed for their association with the occurrence of spinal infections (Table 4) (20, 28, 34–44).

**Table 4:** **Intra-operative factors associated with high risk for SSI (20, 28, 36, 39–44)**.

Surgical invasiveness index
Type of fusion performed
Implants use
Revision intervention (compared to primary intervention)
Traditional open approach (compared to minimally invasive approach)
Site of surgery (dorsal surgeries with highest infective risk compared to cervical and lumbar locations)
Omission of drain usage post spine surgery
Administered fraction of inspired oxygen less than 50%
Operative duration above 3 h
Instrumentation alloy from stainless steel (compared to titanium use)

The assessment of both pre-operative and intra-operative risk factors is mandatory according to best clinical practice. This would aid physicians in their operative approach choices and would allow closer follow-up for high-risk patients. We suggest the prospective development of an SSI risk score that would be inclusive of the relevant risk factors (Tables 3 and 4). There is a need to conduct studies aiming at generating this risk score based on reliable evidence. This score would be used as a tool to limit the ambiguity in the management and follow-up of patients undergoing spine surgery. The NHSN SSI standardized infection ratio (SIR) risk adjustment model can serve as a guide for the development of a specific one for spine surgeries (45).

## Microbial Etiology

Confirming the microbial etiology of SSI following spine surgery is of paramount importance to appropriately guide antimicrobial therapy. This is specifically important in the era of increasing antimicrobial resistance. Empirical antibiotic therapy is highly discouraged before obtaining the proper specimens for cultures (46, 47). Blood and wound cultures are recommended in patients presenting with suspected SSIs. When a deep collection is localized, obtaining a CT-guided specimen for Gram stain and culture is highly recommended. When surgical intervention is indicated and removal of implants is done for curative treatment, vortexing and sonication of the extracted implants to release biofilm-embedded organisms have shown an improvement in the diagnostic yield. Cultures from sonicated spinal implants have reported sensitivity and specificity rates of 93 and 97%, respectively, for confirming the causative pathogen (48).

Surgical site infection following spine surgery usually occurs through direct inoculation during the surgical procedure. The other two possible routes of infection are hematogenous spread and early post-operative contamination. *Staphylococcus aureus* remains the leading agent of SSI responsible for around 50% of cases, although estimates in various studies range from as low as 12–65% (12, 28, 48–50). Additional common causes of SSI include coagulase-negative staphylococci, mainly *Staphylococcus epidermidis*, mostly associated with implanted spinal prosthesis. In addition, Gram-negative organisms can be encountered, including *Pseudomonas aeruginosa, Escherichia coli*, and *Proteus species* (9). Gram-negative organisms are more likely to occur in cases of hematogenous seeding, and in lower lumbar surgical interventions due to proximity to the perianal area. Additional risk factors for SSI with Gram-negative bacteria include bladder or fecal incontinence, a previous history of long hospital admission and a posterior lumbosacral surgical approach (51). A history of intravenous drug use has also been associated with an increased risk of Gram-negative pathogens (9, 52). Recognizing all these risk factors would help in guiding empirical antimicrobial therapy pending culture results.

More recently anaerobic microorganisms such as *Propionibacterium acnes*, part of the normal skin flora, have been increasingly reported to cause various orthopedic-related SSIs including spine surgeries (53–55). *P. acnes* is difficult to detect in patients undergoing spinal surgery with instrumentation (56). Patients with *P. acnes* infection have a typical clinical presentation. They are usually in the fifth or sixth decade of life. They present with low-grade or no fever following a posterior spinal fusion and instrumentation and often present with a late (more than 30 days) post-surgical back pain as a sole complaint (55). Other than its usual indolent and delayed post-procedural clinical presentation, two additional reasons may explain the under-diagnosis of *P. acnes* SSI. First, this organism is considered as a low virulence skin organism, and thus is ignored in some cases to be the causative agent in SSI. Second, *P. acnes* is a slow growing microorganism that requires extended incubation time for growth (52). Thus, when this organism is suspected, several deep tissue specimens should be obtained and cultured for extended periods.

## Diagnosis

**Figure 1:**
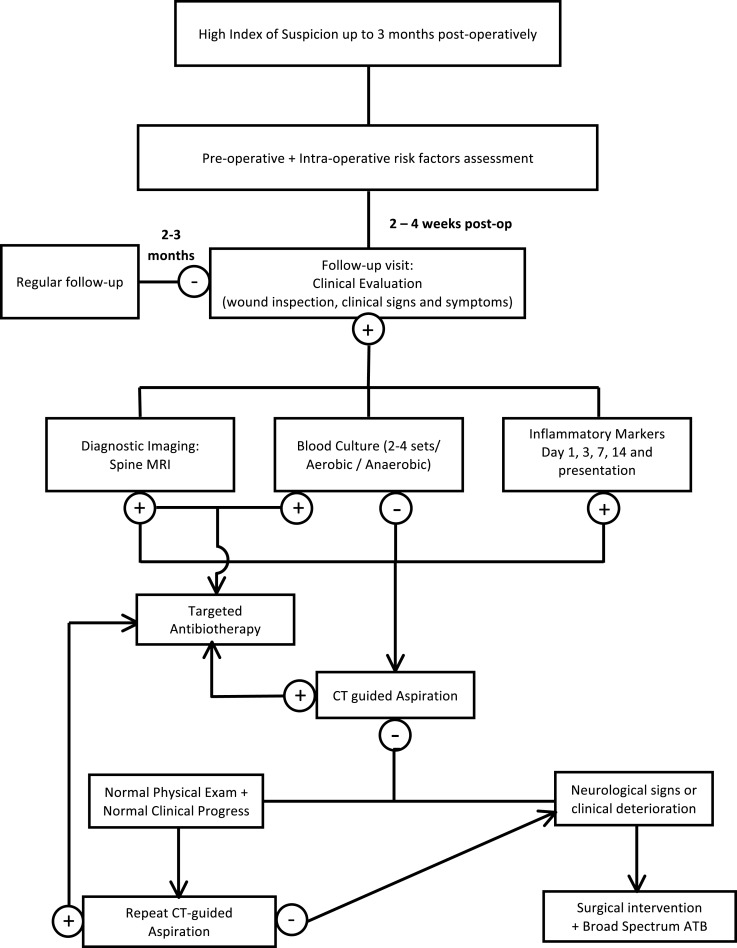
**Algorithm for rapid diagnosis and management of post-surgical spine infections**.

Establishing the diagnosis of SSI can be very challenging, and a high index of suspicion should always be maintained for any patient presenting with back pain within a window period of 3 months after the procedure. Figure 1 represents an algorithm for a diagnostic approach to suspected surgical spine infections and is detailed in the following sections. A number of difficulties may delay the confirmation of SSI. Although the usual duration between the invasive procedure and the occurrence of the infection ranges from 2 to 30 days (57), many indolent organisms like *P. acnes, Serratia marcescens*, diphtheroids, and coagulase-negative staphylococci classically present after 30 days (34). In addition, patients may not seek early medical attention since infection may present initially with minimal back pain as the only clinical finding and the lack of prompt medical follow-up after the procedure may therefore aggravate the problem (28, 52).

There remains a need for large multi-center clinical studies to address the optimal diagnostic approach for SSI. In this section, we will analyze currently available diagnostic modalities and the controversies that surround them.

### Clinical presentation

The most common presenting symptom for SSI following spine surgery is back pain, usually 1 month after the procedure with a range of 2 days to over 3 months post-intervention (57). Studies focusing on spinal procedures with instrumentation showed that the onset of SSI is more delayed in this category of patients, with a mean time to SSI diagnosis of 14 months, thus underlying the need for long-term follow-up subsequent to instrumented spinal procedures (25). The pain is characteristically localized, continuous, and not relieved by pain medications. It can radiate to the hip, leg, scrotum, groin, abdomen, or perineum. It has a slow, insidious onset, which can cause diagnosis to be delayed. It could appear after a pain-free interval for as long as 3 months (29). Other characteristic clinical features are wound drainage and constitutional symptoms such as fever, present in 40% of the cases, fatigue, and weight loss (28, 57).

Additionally, physical examination may reveal localized tenderness, warmth, erythema, and edema at the site of surgery with or without purulent wound drainage (9). Purulent wound drainage occurs in around two-third of SSIs with instrumentation and is the most frequent indicative sign of instrumented spine surgery infections (48).

Deep infections present more commonly with constitutional symptoms, and in rare cases, patients might suffer from severe sepsis and end organ failure. Deep infections often lack impressive superficial features making their diagnosis solely presumptive.

### Inflammatory markers

The most frequently used laboratory tests for both the diagnosis and follow-up of patients suspected to have SSI following spine surgery are: white blood cell count (WBC), differential erythrocyte sedimentation rate (ESR), and C-reactive protein (CRP). The WBC is elevated in less than 50% of SSI cases, thus making it an unreliable diagnostic marker (58). Although CRP and ESR are highly sensitive in the detection of any SSI, many barriers face their use in daily practice. ESR and CRP levels increase post-operatively rendering the differentiation between infected and non-infected patients problematic in the early post-operative window period. In fact, CRP levels possess low positive predictive values (31%) in the diagnosis of SSI (59). For instance, CRP levels peak on day 3 and decrease to normal baseline between days 10 and 14 post-operatively (60). On the other hand, ESR levels are highest at around 14 days and do not normalize until approximately 6 weeks after surgery. The earlier normalization of CRP and its higher sensitivity in diagnosing SSI compared to ESR (95 vs. 80%) make it a more useful tool (60–64). In cases of suspected SSI, it would be very useful to compare CRP levels at day 7 to those on day 3; a detected elevation on day 7 would raise the suspicion for an infective process.

Some factors have been reported to influence CRP level. These include the amount of blood loss, the pre-operative CRP level, and the location of spinal intervention. For example, surgeries on the lumbar region were found to be associated with higher post-surgical levels of CRP compared to procedures involving other regions (62).

In view of these uncertainties surrounding the use of inflammatory markers as diagnostic tools for SSI, additional tools are desirable to distinguish between infected and non-infected patients in the post-operative period.

Serum amyloid A (SAA), conventionally considered for its role in the pathogenesis of amyloid A-type amyloidosis, has been recently studied for its immunological activities, specifically its function in activating Toll-like receptor (TLR) 2 and TLR4, class B scavenger receptor CD36. A study by Deguchi et al. compared the serum levels of CRP and SAA after posterior approach spine surgery. The authors found that both markers achieved their highest levels on day 3. Although the levels later decreased, the rate of normalization of SAA was faster compared to CRP. SAA has a short half-life of 50 min compared to 5–7 h for CRP. This distinctive rapid decrease in SAA in non-infected cases is very helpful in eliminating one of the controversies surrounding the diagnosis of early SSI (65). Another remarkable finding in this study was that with the administration of corticosteroids, while the serum level of CRP decreases or even normalizes, that of SAA is not altered. Additionally, SAA levels did not vary with gender or age, and the marker preserved reactivity in patients with rheumatoid arthritis receiving corticosteroid therapy. Therefore, SAA can be considered a better inflammatory marker in the assessment of SSI following spine surgery (65).

Other inflammatory markers such as procalcitonin have been less promising in the diagnosis of SSI (66). One study compared the level of procalcitonin in 17 patients with spondylodiscitis and 18 patients with herniated disks and found no elevation in either group.

Our recommended approach for a comprehensive follow-up of patients undergoing invasive spinal procedures is using inflammatory markers with a combined comparative measurement of WBC, CRP, and SAA pre-operatively, at day 1, 3, 7, and 14 and upon presentation with suspected SSI (65).

### Imaging

Imaging is considered a key element for the diagnosis and follow-up of SSI following spine surgery. Even though magnetic resonance imaging (MRI) remains the technique of choice, plain X-ray has always been the first to be ordered when suspecting an SSI. However, this imaging lacks sensitivity in detecting SSI, which can cause delays in the establishment of a definitive diagnosis (12, 67). In fact, post-operative plain radiographs performed up until 4 weeks post index procedure, are expected to show normal or unchanged spinal structure compared to pre-operative images (2, 67). The first pathologically defining change to appear between the fourth and sixth weeks post-operatively is a decrease in intervertebral height. Other plain radio film manifestations, including osteolysis, deformity, and endplate destruction, are only expected to appear after 6 weeks (2, 67).

As for CT scanning, interest was lost in this technique after the advent and widespread availability of MRI. However, CT scan imaging can be used to assess bony destruction and spinal stability with great precision (2, 9). It can aid in planning transcutaneous aspiration and in the surgical approach and technique.

Radionuclide imaging has shown higher sensitivity and earlier detection of SSI compared to CT scanning and X-ray imaging. For instance, Gallium 67 scanning can show focal increased uptake areas suggestive of infection with high sensitivity and specificity of 89 and 85%, respectively (2, 68, 69). Moreover, it is estimated that the interval for the appearance of diagnostic radiological signs of SSI is shortened with Gallium 67 compared to Technetium 99 scanning (2, 68, 69), rendering Gallium 67 the preferred agent for early detection of SSI radiological changes.

Magnetic resonance imaging is currently the most appropriate imaging to be performed as a first step when suspecting SSIs (70, 71). MRI is both the most sensitive (93%) and specific (96%) technique to evaluate SSIs following spine surgery (72, 73). The diagnostic features of SSI can be detected as early as days 3–5 post-operatively. Characteristic findings include diminished disk height, vertebral body, and disk space, decreased intensity on T1-weighted images, increased signal intensity on T2-weighted imaging secondary to edema, and endplate definition loss. These vertebral disk changes are accompanied by increased bone marrow intensity signaling due to edema (74).

Early in the infection process, short tau inversion recovery (STIR) sequences offer higher signal intensity to help differentiate the infected area from the normal spine, but with a drawback of inferior anatomic detail (73). Although MRI remains the imaging of choice, many limitations hamper its role in the diagnosis of SSI. Diagnostic findings depend on many technical parameters, and artifacts from implants may hinder the interpretation of results (76, 77). In addition, some degenerative or inflammatory non-infectious diseases may simulate spinal infection leading to a false positive result (75, 76). Moreover, an important limitation involves the follow-up of patients with SSIs. There is often no noted difference in the MRI between patients who have shown clinical improvement and those who did not (77). Thus, the clinicians’ interpretation of follow-up MRI results should focus on soft tissue rather than bony findings, keeping in mind that no single parameter in MRI results is significantly correlated with the clinical status variation (77).

Many studies have been recently undertaken to define the imaging technique that will resolve the above-mentioned controversies and limitations surrounding the radiological diagnosis of SSI following spine surgery. 18F-fluorodeoxyglucose-positron emission tomography (FDG-PET) imaging is one modality that has lately shown utility in patients with suspected SSI (76). In a prospective cohort study that assessed over 300 patients, Ohtori et al. found that a definitive diagnosis of SSI was achieved more often when 18FDG-PET was utilized (78). This was reported specifically in patients with spinal infections presenting as Modic type 1 signal on MRI, which made the distinction between common Modic changes and SSI challenging (78, 79). The benefit of 18FDG-PET imaging is its usefulness in the work-up of patients with metallic implants, since it is not affected by artifacts. In fact, 18FDG-PET imaging is characterized by high sensitivity and specificity and can provide results within 2 h with a resolution of up to 4–5 mm. In addition, it entails a relatively low exposure to radiation and helps in distinguishing between initial spondylodiscitis and degenerative changes in the vertebral body endplate (79). One limitation of this technique is that it is difficult at times to distinguish an infectious process from a malignancy (80). However, in the right clinical setting, this is not expected to be an issue in the post-operative period. Unfortunately, due to its high cost and limited availability, the use of this technique remains limited, and it is indicated as a second line option for complicated cases with significant delay in the establishment of a definitive diagnosis, or when MRI is contraindicated (78–81).

### Etiological diagnosis

The isolation of a specific pathogen is of crucial importance in the diagnosis and management of SSIs following spine surgery. Blood cultures (two to four sets) constitute the simplest procedure to detect the pathogen. When the blood culture yields a highly pathogenic organism such as *S. aureus, P. aeruginosa*, and no other sites of infections are obvious, then this is diagnostic of the causative agent. However, blood cultures lack both sensitivity and specificity in detecting the pathogen (82).

Superficial infections do not pose significant controversies and their diagnosis is more obvious than that of deep infections. In superficial spine infections, Gram stain and culture of wound swabs or aspirated fluid are recommended. Although very easy to perform, wound swab cultures lack specificity due to contamination by normal skin flora. In contrast, deep-seated spine infections require CT or fluoroscopic-guided deep aspiration from the infected area. This has shown a low diagnostic yield of around 40% when compared to cultures from intra-operative infected tissue, which remains the most reliable method of pathogen detection. Previous use of antibiotics is the main reason for the low diagnostic yield of fluoroscopic-guided aspiration (82–85). However, since the procedure has already been established to be both safe and minimally invasive, it should be done in all patients with deep-seated infections with negative blood cultures.

Three main recommendations may help in improving the diagnostic yield and avoiding false negative cultures. First, the use of a large bore needle is encouraged (86). Second, histopathological examination should be requested as an adjunct to cultures if adequate tissue can be safely obtained, since this can help identify pyogenic from granulomatous changes in tissues. The use of additional staining for fungi and mycobacteria is highly advised, especially with previous negative culture results (87–90). Third, a delay in antibiotic therapy is recommended in order to achieve a better culture yield of aspirated specimens (52). This should be balanced with potential consequences of delayed treatment. In patients with normal neurologic examination and normal hemodynamic status and who had been started on antibiotics before the performance of CT-guided aspiration, withholding therapy for 1 week before performing the procedure is advisable. However, for patients with neurological compromise, broad-spectrum empiric antibiotic therapy and immediate surgical intervention are warranted (91).

These recommendations are intended to maximize the yield of cultures obtained from CT-guided aspirations. However, if cultures are negative, the patient’s neurological and hemodynamic status is of pivotal importance in determining the best next step in clinical practice. Repeating the percutaneous aspiration is recommended if the patient has a normal neurological evaluation, is hemodynamically stable, and has no major underlying morbidities (92, 93). Conversely, in patients who present with deteriorating general or neurological status, rapid intervention is crucial to improve patient outcome. Surgical intervention is recommended, along with obtaining cultures of intra-operative specimens, since this has shown increased diagnostic yield even if minimally invasive techniques had been used for sampling (94–96).

Two techniques have improved the yield of microbiologic confirmation of the offending organism. First, the use of novel DNA microarrays and polymerase chain reaction (PCR) molecular amplification techniques of aspirated or sampled tissues and fluid during surgery or percutaneous aspiration and biopsy have shown high sensitivity and specificity and could be instrumental in defining the etiological diagnosis of SSI (97–102). This technique offers a particular advantage in patients with prior antibiotic use. In addition to classical bacterial pathogens, PCR amplification can effectively detect mycobacterial species. Given the extremely elevated morbidity and neurological dysfunction associated with untreated spine infections, these molecular techniques may help in obtaining a quick definitive diagnosis of SSI and prompt an earlier initiation of targeted antibiotherapy (97–102). However, susceptibility testing cannot be obtained through molecular testing, highlighting the superiority of cultures.

Second, vortexing and sonication of retrieved spinal implants to disrupt the biofilms has been recommended. Even though this technique is not widely available for daily practice, it has shown a sensitivity of 93% and a specificity of 97% in the isolation of pathogens involved in SSIs following spine surgery (48). Additional efforts are needed to increase availability, accessibility, and utilization of these new methods (48).

## Conclusion

Surgical site infection following spine surgery is a major cause of increased morbidity following spine interventions and an immense burden on the health care system. A high index of suspicion should be kept in the first 3 months after the procedure. The diagnosis is usually suspected based on the symptoms and physical exam findings. However, a number of laboratory and imaging techniques are available to speed up the confirmation of the diagnosis and the recovery of the offending organisms, which would allow early targeted therapy.

## Conflict of Interest Statement

The authors declare that the research was conducted in the absence of any commercial or financial relationships that could be construed as a potential conflict of interest.
